# Mapping the Landscape of the Digital Workflow of Esthetic Veneers from Design to Cementation: A Systematic Review

**DOI:** 10.3390/dj12020028

**Published:** 2024-01-31

**Authors:** Walaa Magdy Ahmed, Amr Ahmed Azhari, Lamer Sedayo, Alanod Alhaid, Reem Alhandar, Amirah Almalki, Aishah Jahlan, Afnan Almutairi, Waad Kheder

**Affiliations:** 1 Department of Restorative Dentistry, Faculty of Dentistry, King Abdulaziz University, Jeddah P.O. Box 80213, Saudi Arabia; aaaazhari@kau.edu.sa; 2 Faculty of Dental Medicine, Umm Al-Qura University, Makkah P.O. Box 16786, Saudi Arabia; 3 Dental Department, King Khalid University, Asir P.O. Box 61421, Saudi Arabia; 4 Dental Department, Vision College, Riyadh P.O. Box 13226, Saudi Arabia; 5 Dental Department, Specialized Dental Complex, Jeddah P.O. Box 21444, Saudi Arabia; amirah.a.am95@gmail.com; 6 Department of Restorative Dentistry, Suliman Al-Habib Hospital, Riyadh P.O. Box 301578, Saudi Arabia; 7 Department of Restorative Dentistry, College of Dental Medicine, University of Sharjah, Sharjah P.O. Box 27272, United Arab Emirates; wkheder@sharjah.ac.ae

**Keywords:** esthetic veneer, digital dentistry, esthetic dentistry, accuracy, preparation guide, cementation guide, digital smile design

## Abstract

The purpose of this systematic review was to map all the existing literature on digitally designed and fabricated esthetic veneers. We aimed to compare the accuracy of digitally designed preparation and cementation guides for esthetic indirect veneers with the conventional workflow. We evaluated studies comparing the accuracy and predictability of workflows between digitally fabricated indirect-esthetic veneers and conventional indirect veneers. Searches were performed in August 2023 across three databases, specifically Google Scholar, Cochrane, and PubMed, and were restricted to English-language publications. The search strategy was based on the PICO criteria. Reference lists of identified articles were manually checked to find further pertinent studies that were not discovered during the electronic search. The titles and abstracts were reviewed in the first stage, and then the full article texts were reviewed and cross-matched against the predetermined inclusion criteria. Following the search, 169 articles were identified: 41 from Google Scholar, 44 from Cochrane, and 71 from PubMed, with 13 added manually. Of these, 20 were chosen for a detailed quality assessment of the digital veneer workflow and the accuracy of digital preparations and cementation guides for laminate veneers. Based on our findings, the digitally fabricated laminate-veneer workflow demonstrated superior predictability and accuracy compared to the conventional workflow.

## 1. Introduction

Esthetic dentistry is one of the most dynamic areas in contemporary dentistry [1]. Ceramic laminate veneers represent a minimally invasive restoration technique that combine nature and beauty, offering superior esthetics [1,2]. These veneers are thin, bonded-ceramic restorations that cover the facial, incisal, and a portion of the proximal surfaces of teeth in need of esthetic restoration [3]. The success and longevity of veneers rely primarily on the minimal preparation of the enamel. This aims to decrease stress and facilitate proper cementation with adhesive luting materials for retention and strength [2,4]. However, achieving an ideal preparation within millimeter fractions is challenging [5]. Due to the introduction of digital technology, veneer preparation and bonding have become more streamlined, aided by 3D-printed guides [6]. The use of design–computer-aided manufacturing CAD/CAM innovations to design and fabricate restorations is a predictable and time-efficient procedure [7]. However, the skills and clinical experience of technicians remain essential [8].

Ceramic veneers are restorations that provide a high level of esthetic appeal and demonstrate consistent and predictable results [9]. When bonded to enamel, they exhibit exceptional success rates [10,11,12,13,14,15,16]. Nevertheless, it is worth noting that, as the extent of tooth structure removal grows, there is generally a decrease in the success rate of the restoration, accompanied by an escalation in biomechanical risk. Digital smile design protocols allow clinicians to visualize the final tooth position and shape before committing to irreversible changes [17]. The outcomes of restorative procedures in the esthetic zone depend on clear communication between the patient and dentist, coupled with an accurate diagnosis and treatment plan. Over time, restoration planning for esthetically demanding cases has evolved from wax-up diagnostic casts to virtual designs created from digital photographs, aided by lines and rulers [13,17]. However, wax-up diagnostic casts and mock-ups are crucial to clarify the suggested modifications in restored teeth and obtain patient approval.

The process of tooth preparation for porcelain laminate veneers (PLVs) entails the simultaneous removal of the tooth structure to achieve the desired porcelain thickness and the preservation of the tooth structure to ensure appropriate retention. Hence, it is imperative to ensure accurate tooth preparation prior to the placement of PLVs. Tooth preparation guides, when used collectively, possess the ability to produce accurate and exact tooth preparation for PLVs. In addition, it should be noted that conventional freehand PLV cementation processes exhibit a high degree of technical sensitivity. Potential errors can arise during several stages of the restoration process, particularly in the preconditioning phase and the fitting of PLVs using finger pressure. These errors have the potential to negatively impact the final outcome of the restoration. The utilization of repetitive bonding procedures in PLVs has been found to elevate the potential for problems, resulting in prolonged chairside durations and suboptimal patient satisfaction.

This study was conducted to evaluate the workflow for digital veneers and the precision of digitally designed preparation guides for laminate veneers compared to the conventional workflow. This evaluation focuses on achieving precise tooth preparation with minimal enamel removal to optimize bonding. We also assessed the accuracy of digitally designed cementation guides for laminate veneers in contrast to the conventional workflow, as this could help prevent issues like microleakage, cement dissolution, and other complications. Ultimately, this research seeks to present a comprehensive systematic review on digitally designed preparation and cementation guides for laminate veneers. The reasons that led to developing this study stems from the recognition that digital dentistry is rapidly transforming clinical practice. Conducting this review to evaluate the available evidence regarding integrating emerging technologies provides an understanding of the best practices and consensus on the digital workflow for veneer fabrication and placement.

Therefore, the aims of this systematic review are as follows: (1) mapping all the available literature on digitally fabricated ceramic laminate veneers, (2) evaluating the accuracy of digitally designed preparation guides for laminate veneers against the conventional workflow, and (3) assessing the precision of digitally designed cementation guides for laminate veneers in comparison to the conventional approach. The null hypotheses were as follows: (1) There will be no difference between the fabrication of digital versus conventional laminate veneers. (2) There will be no significant difference between the accuracy of digitally designed preparation guides for laminate veneers and those for conventional veneer preparations. (3) There will be no significant difference between the accuracy of digitally designed cementation guides for laminate veneers and that of conventional veneer cementation.

## 2. Materials and Methods

### 2.1. Protocol and Registration

This review protocol was registered in the PROSPERO database under the record ID 507107.

### 2.2. Eligibility Criteria

The search strategy was determined according to the PICO format. We included both in vitro and in vivo English-language, peer-reviewed studies addressing the workflow for digital veneers and those comparing the conventional veneer workflow to the digital workflow. Studies that evaluated the accuracy of the workflow for digital and conventional veneers were included. The search was narrowed by excluding studies on composite veneers, direct veneers, inlays, onlays, endodontic crowns, occlusal veneers, and overlays. All review articles and non-English publications were also excluded. We excluded articles that solely discussed the conventional workflow for veneers or those addressing dental cast accuracy, bond strength, impression systems, and digital impressions.

### 2.3. Search Strategy

The search strategy was determined according to the “Population, Intervention, Control, and Outcome” PICO model using Google Scholar, Cochrane, and PubMed. Population is the esthetic veneers, Intervention is the digital workflow, Control is the conventional workflow, and Outcome is the accuracy.

During the systematic search, keywords such as (“laminate veneer” OR “porcelain veneer” OR “luminaire” OR “translucent veneers” OR “ceramic veneers” OR “indirect veneer” OR “digital veneer”) AND (“digital workflow” OR “guide*” OR “preparation guide” OR “cementation guide” OR “reduction guide” OR “smile design” OR “intraoral scan” OR “CAD/CAM”) AND (“conventional” OR “traditional” OR “freehand” OR “smile makeover”) AND (“accur*” OR “accuracy” OR “precision” OR “trueness” OR “esthetic” OR “esthetic result”) were input.

### 2.4. Selection Process

**Figure 1:**
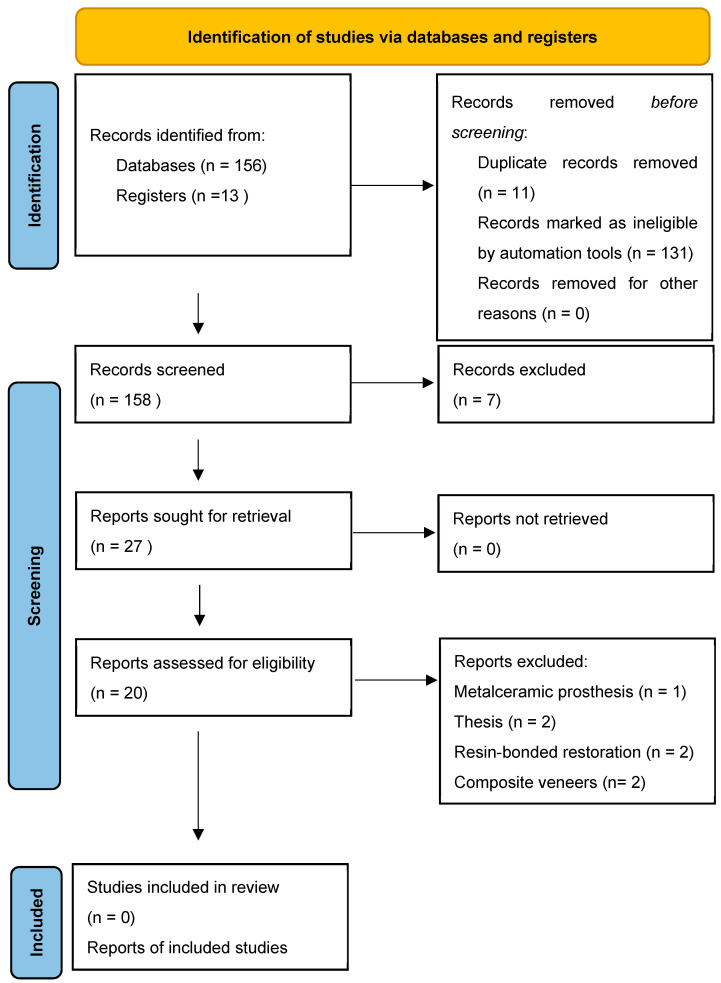
PRISMA 2020 [18] flow diagram for article selection and inclusion in the systematic review.

The reference lists of the related articles were manually checked for supplementary pertinent articles that were not discovered during the electronic search. Two of the reviewers (LS and AA) independently applied the inclusion and exclusion criteria for the selections. Initially, articles were chosen based on their titles and abstracts. This was followed by a review of the entire text of the shortlisted articles. In instances in which the reviewers disagreed, they consulted a third author (AJ). The consistency between the abstract and full-text selections made by different reviewers was assessed using Cohen’s kappa coefficients (0.86). The search strategy was adjusted as per the specific requirements of the database in question. The initial search yielded a total of 169 articles: 41 from Google Scholar, 44 from Cochrane, 71 from PubMed, and 13 studies that were manually included. After eliminating duplicates, the titles and abstracts of the initial set of articles were reviewed. Subsequently, the full texts of the articles were reviewed and cross-matched against the predetermined inclusion criteria (Figure 1).

### 2.5. Certainty of Evidence

The interobserver calibration was assessed using Cohen’s kappa statistic with a predetermined threshold of 80%. The researchers used the GRADE criteria to provide a framework for evaluating the quality of the papers chosen for analysis [19,20]. The quality was classified as high (H), moderate (M), low (L), or very low (VL). Quality pertains to the level of certainty of the accuracy of the estimated impact. According to Balshem et al. (2011), the GRADE approach distinguishes between the evaluation of the evidence quality and the development of recommendations. Decisions regarding the formulation of guidelines are influenced by factors beyond the mere quality of evidence.

## 3. Results

The electronic search yielded a cumulative count of 169 articles: 41 from Google Scholar, 44 from Cochrane, and 71 from PubMed. In addition, 13 articles were selected for the manual search. Initially, the titles and abstracts of all the articles were reviewed. Subsequently, the full text of all 169 articles was assessed and compared against the predetermined inclusion criteria. In the end, only 27 articles were shortlisted. Adhering to the exclusion criteria, out of these 27, only 20 articles were finalized for the quality assessment of the workflow for digital veneers and the accuracy of digitally designed preparation and cementation guides for laminate veneers. Tooth preparation guides are expected to provide a more accurate veneer preparation procedure than freehand preparation. Veneer cementation guides are expected to provide a more predictable, accurate, and efficient simultaneous preconditioning and cementing of PLVs in contrast to traditional cementation (Table 1). The reasons for the exclusion of articles after full-text reading are presented in Table 2.

## 4. Discussion

**Figure 2:**
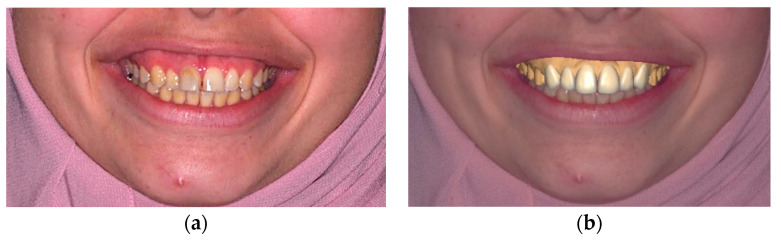
(**a**) Pre-operative frontal view photograph without DSD, and (**b**) pre-operative frontal view photograph with DSD.

The workflow for esthetic veneers encompasses multiple stages, beginning with case selection and progressing to digital smile design, guided tooth preparation, and the taking of scans or impressions for final prosthesis fabrication, followed by the bonding protocol and finalizing with maintenance and follow-up scheduling. The conventional technique for smile design holds a higher potential for human error, considering its lack of facial orientation. It predominantly relies on the technician’s manual skills and experience to develop a plan based on radiological and clinical examinations, intra- and extraoral analyses, occlusal evaluations, and impressions [17,40]. However, digital smile design is facially oriented either in 2D versions, using numerous photographic editing tools, or in 3D versions, where the intraoral scanner is superimposed on the face scan or photos before designing the desired features (Figure 2). This approach enables operators to graft the tooth structure, bridge gaps between teeth, adjust the smile line, and alter the tooth color within a facially oriented frame. However, post digital smile design, a manual diagnostic wax-up on the calibrated stone model is still required. Additionally, transferring data from the virtual design to the stone model poses challenges and opportunities for potential error [17].

Alshali and Asali (2022) showed that digital smile design using two different software packages and feldspathic porcelain for restoring anterior maxillary teeth with porcelain laminate veneers demonstrates an effective and predictable workflow, leading to adequate esthetic results as compared to the conventional workflow [24]. The significance of this particular case is that the authors and patient agreed that the conventional technique yielded better results. This could be because the technicians’ skills were evident in the wax-ups and final restorations. The optical properties of feldspathic porcelain compared to those of milled Emax could be another reason for these results. In addition, in this case, there was no dark staining or discoloration of the teeth, which might require a more aggressive teeth preparation and/or a higher-opacity material. In 2018, Lin et al. confirmed this, observing no complications 6 months post-insertion of veneers crafted from machinable lithium disilicate ceramic blocks and utilizing digital smile design [30]. This indicates the accuracy of the digital smile design in fabricating porcelain veneers. However, case complexity and operator experience played significant roles in both studies. Furthermore, using the technique of mock-up design and fabrication is crucial for the final results of ceramic veneers. The use of smile-designing software facilitates interdisciplinary collaboration between practitioners, which seems to improve the decision-making process and ultimately decrease the number of intraoral adjustments. This tool allows the patient to preview the prosthetic result directly on a picture and provides the dental technician with all the necessary information regarding the execution of the work through a detailed report. Facial scans and intraoral scans are essential tools for creating a 3D virtual patient. However, the high cost of these scans can be impractical for dental clinicians. Sense, a cost-effective extraoral 3D scanner, is the preferred option. Combining these scans requires extra time, resources, and training for dental laboratory technicians. Additionally, it results in additional expenses for both clinicians and patients. To improve accuracy, clinicians should ensure consistent facial expressions and head positioning during the scanning process.

In 2020, Lo Giudice et al. conducted an in vivo study and found that both the prototype and milled mock-ups exhibited a slight dimensional augmentation compared to the original 3D project. Interestingly, the milled mock-ups demonstrated a diminished fit after clinical tests [26]. It is essential to approach the trueness of scanned manufacturing with caution, as inherent system errors might lead to the underestimation of the actual object dimensions. The minimal amount of material required in 3D printing for prosthesis manufacturing is well-suited to clinical adaptation. This method enables multiple products to be created simultaneously, enhancing efficiency. However, the study’s small sample size and use of a single milling machine and 3D printer should be interpreted cautiously. Further ex vivo and in vivo studies with larger sample sizes and using different milling and prototyping technologies are required. Similarly, an in vitro investigation conducted by Cattoni et al. in 2019 revealed accuracy discrepancies between the traditional molded and milled mock-ups versus their original wax-up casts [17]. Statistical research revealed that the utilization of the digital approach yielded higher levels of precision. Considering the constraints of Cattoni et al.’s research, a completely digital method is seen as more dependable for producing an aesthetically pleasing mockup. The digital technique has demonstrated superior accuracy compared to manual execution, which is considerably reliant on the operator and raises the likelihood of errors; this could ultimately impact the final outcome.

There is also a case report by Ryan Tak On Tse regarding merging clear aligner therapy with a digital mile design to maximize esthetics and minimize tooth reduction [41]. Utilizing DSD enables clinicians to strategically prepare different treatment techniques and visualize the anticipated results before the actual surgery. The integration of Invisalign and DSD allows the dentist to effectively reposition the teeth to obtain optimal alignment for veneer repair, resulting in the desired esthetic outcomes while using minimally invasive dental procedures. The following veneers seamlessly conform to the face structure and lower lip, with gaps expertly repaired. The utilization of the additive methodology in this instance not only minimized the amount of tooth preparation, but also enhanced the appearance of the teeth in the patient’s smile. The patient conveyed contentment with the outstanding esthetics and the cautious approach to the extraction of the tooth structure. The incorporation of clear aligner therapy with DSD provides precise guidance and assessment in straightforward circumstances. An efficient and cost-effective method for carrying out this merger is the three-line technique, which effectively demonstrates the symmetry and angulation of peg laterals, precisely strategizes the repair of crossbites, and minimizes the removal of tooth material from the central incisors. Nevertheless, the margin of error continues to provide a barrier until it is fully included.

Bruno Pereira da Silva’s case study highlights the benefits of digitally guided tooth preparation for laminate veneers [6]. The technique for preparing veneers provides a less invasive approach and uses digital guidance, which can improve accuracy, efficiency, and predictability. Additionally, it reduces the amount of time the patient spends in the dental chair. Nevertheless, the two-step method may incur supplementary expenses, such as the acquisition of a new handpiece and the production of 3D-printed guidance. Notwithstanding these difficulties, digital technology has the potential to improve therapeutic results and reduce errors in the process of preparing, designing, and administering treatment. Vertical preparation may have limitations in non-additive scenarios or cases with undesirable color substrates, but it grants laboratory technicians and physicians the flexibility to select restoration margins and conserve enamel in the cervical area.

Another study was conducted to assess the accuracy of three digital restorations fabricated via the digital mock-up of ceramic veneers [2], and the results revealed that the trial restoration was significantly thicker than the corresponding waxing. Among the three protocols examined, the DC technique provided the most accurate reduction result. Some patients with protruding teeth are unsuitable for or unwilling to undergo orthodontic treatment, which leads to decreased treatment satisfaction. This inconsistency in trial restoration fabrication may have resulted in differences between waxing and the trial restorations at the cervical site. Consequently, the inconsistency between waxing and trial restorations, possibly leading to inaccuracies during the preparation, should be considered. Inadequate preparation at the cervical site might compromise the strength of ceramic materials or result in an overcontoured restoration, affecting both esthetics and periodontal health. A limitation of this study is its reliance on a software program to gauge the two-dimensional distance at several sites on the tooth’s surface. Such data do not encapsulate the comprehensive context of a three-dimensional preparation. Thus, research employing a digital technique capable of rapidly and automatically measuring the entire tooth preparation is recommended.

**Figure 3:**
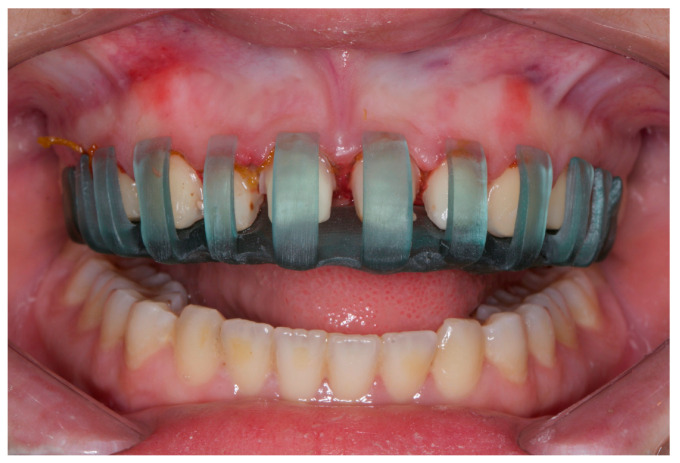
The 3D-designed and printed laminate veneer preparation guide.

Tooth preparation guides provide a more accurate veneer preparation than freehand preparation (Figure 3). A study comparing freehand preparation and tooth preparation guides found that 3D-printed auto-stop guides were more accurate than freehand ones. The study found that the 3D-printed auto-stop guide had the lowest absolute difference in preparation (0.12–0.16 mm), while the silicone guide had the highest absolute difference (0.12–0.16 mm) [5]. The study also observed that veneer preparations were conducted on acrylic teeth within a phantom head to replicate the real-life clinical setting. Nevertheless, the study also observed that trimming softer plastic teeth could potentially compromise the guiding function of the guide. Notwithstanding these constraints, the 3D-printed auto-stop guide was determined to be the most reliable and precise instrument.

The authors of this study conducted a clinical trial on hybrid laminate veneers, examining the survival rates of various tooth preparation techniques. They found that polymer-infiltrated ceramic network laminate veneers showed successful clinical performances in terms of crack formation, secondary caries, endodontic complications, anatomical form, and veneer retention [11]. Despite the outcome monitoring period being short and the majority of participants being female, the color-matching requirements of the VITA ENAMIC laminate veneers deteriorated over time. This study builds upon previous research and suggests that this therapy can be provided by practitioners outside the original intervention team.

There are two techniques able to facilitate the process of cementation in laminate porcelain veneers simultaneously: making the pre-treatment of the ceramic inner surface facile and reducing the time spent sitting in a chair [4,8].

The Chen technique is characterized by a design with lingual perforations that facilitates the process of removing excess cement and verifying the complete separation of PLVs. In contrast, the Silva technique allows the clinician to adjust the veneers while seating them. This ensures that the gingiva’s health is not compromised due to excessively deep gingival margins in the gingival sulcus or the insufficient removal of excess resin cement.

All-ceramic restorations require both internal and marginal adjustments for long-term success. Marginal inadequacies can lead to tooth and periodontal issues, as well as gingival inflammation and plaque retention. Proper marginal fit can help remove excess resin luting agents and minimize the risk of micro-leakage and micro-fracture. Increased internal gaps and cement thickness can chip all-ceramic crowns, reducing their fracture strength. Continuous internal fit enhances seating ease and prevents restoration from occurring or resisting. Traditional cements have an internal spacing of 50–100 m, while adhesive cements have an internal gap of 200–300 m. Research on all-ceramic restorations supported by implants is limited, but metal-ceramic and all-ceramic implant-supported restorations have mean marginal gaps of 11–67.4 m and 58–168 m, respectively. The accurate measurement of marginal gaps requires fixed specimen positions and the same measuring angle. The use of a digital workflow in managing esthetic cases enhances treatment predictability and increases the survival and success of restorations due to the conservation of tooth structure [42,43].

**Figure 4:**
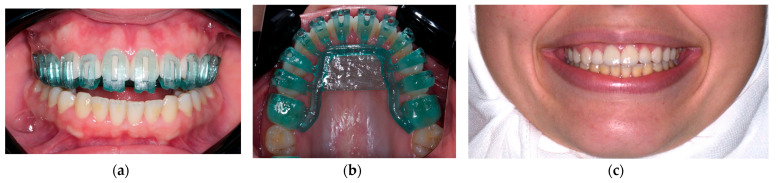
The 3D-designed and printed laminate veneer cementation guide: (**a**) Frontal view, (**b**) occlusal view, and (**c**) after cementation.

This review confirmed that precise tooth preparation with minimal enamel removal attain enhanced bonding, prevent microleakage, reduce the dissolution of cement, and mitigate other complications which is in accordance with other previous studies. Furthermore, a systematic review and meta-analysis of digitally designed preparations and cementation guides for laminate veneers were conducted. One of the limitations of our study was that most of the published studies were either case reports or technical papers that provided a low level of evidence. It is also worth noting that our research was based solely on three databases for the identification of potentially eligible studies. The high variability among the studies limited our ability to conduct a meta-analysis. Specifically, only two studies were related to preparation, four were related to smile design and mock-up fabrication, one addressed the adaptation of digitally fabricated veneers, and another was related to cementation guides (Figure 4).

The motivation for conducting this study arises from the acknowledgement that digital dentistry is swiftly revolutionizing clinical practice. This review assessed the available data on the integration of developing technologies in order to get insight into the most effective methods and consensus on the digital workflow for veneer manufacture and placement. It also assessed the advantages and disadvantages of traditional and digital workflows, providing valuable educational material for practitioners at all levels of experience who are transitioning from conventional to digital methods. The goal is to ensure that they have a strong understanding of the fundamental principles and techniques involved. Systematically mapping the current landscape allows us to identify gaps in the literature where further research is needed to guide future studies and technological developments. By providing a synthesis of the available evidence, practitioners can make informed decisions about incorporating digital workflows into their practice, ultimately enhancing patient care.

## 5. Conclusions

Within the limited available evidence, esthetic veneers obtained from digital smile design showed superior outcomes in term of effectiveness and predictable workflow compared to the conventional workflow. Tooth preparation guides provide a more accurate veneer preparation than freehand preparation. Clinical studies are needed to quantitatively evaluate the efficacy and accuracy of techniques for the fabrication of porcelain veneers. Likewise, there are an insufficient number of randomized clinical trials, studies addressing marginal and internal adaptation, and in vitro and in vivo studies that verify and validate these techniques.

## Figures and Tables

**Table 1:** Summary table of studies included in the systematic review.

Study		Aim	Design	Description	Outcome	Quality Assessment	Limitation
[21]	Workflow	To elucidate a cutting-edge methodology for achieving a smile transformation through the utilization of a completely digitized process.	Case report	This clinical report outlines a methodology for achieving a smile makeover through the utilization of a comprehensive digital veneer workflow. This process involves the creation of 3D-printed smile design models, which are subsequently employed in the clinical setting to guide veneer preparation and provisionalization procedures.	Implementing a digital workflow in the management of esthetic situations improves the ability to predict treatment outcomes and boosts the longevity and effectiveness of restorations by preserving tooth structure.	High	No cementation guide. No mention ofrubber dam isolation during cementation.
[22]		This case report describes an ultraconservative approach that involves the use of only two ceramic laminate veneers for the maxillary central incisors. The purpose of this approach is to improve the patient’s smile.	Case report	The procedure employed adhered to a meticulously designed sequence, which encompassed a diagnostic mock-up, limited tooth reduction guided by a reduction guide, the creation of esthetically pleasing hand-crafted veneers, and the application of rubber dam isolation during the bonding process.	The findings indicate that employing this highly conservative method can greatly improve the appearance of smiles while maintaining the integrity of the original tooth structure. This therapy option is a superior alternative to more intrusive procedures and typically leads to a high level of patient contentment.	High	No cementation guide.
[23]	Workflow	To introduce a predictable digital workflow for minimally invasive anterior-esthetic tooth rehabilitation based on the global diagnosis principle.	Case report	Extra/intraoral photos + intraoral scan → approved 3D smile design. Intraoral scanning was used to record the final preparations. Smile design was superimposed on the preparation to design the final prosthesis.	The digital technique enabled predictive treatment for esthetic rehabilitation by generating a virtual digital patient using the principles of global diagnosis. The treatment is characterized by its minimal invasiveness, which facilitates diagnosis, improves communication, reduces the processing time, and enhances the predictability of outcomes. Additionally, it provides a high comfort level and achieves esthetically pleasing results.	Moderate	Quantitative measurements after superimposition are missing.
[24]	Smile design/Mockup	To outline a systematic treatment process for the restoration of anterior teeth using PLVs, encompassing both traditional and digital techniques.	Case report	This clinical report described the utilization and comparison of two digital smile design software tools, GPS, and Nemo DSD 3D, with the conventional workflow.	The digital smile design programs demonstrated an effective and predictable workflow, resulting in satisfactory esthetic outcomes. Nevertheless, the conventional workflow yielded conservative and esthetically superior results. Feldspathic veneers were chosen for their superior optical properties as compared to CAD/CAM lithium disilicate.	Moderate	No cementation guide used.
[25]		To propose a method for implementing guided tooth preparation in which the tooth is virtually prepared in a laboratory setting, and then preparation templates are developed for use during chairside procedures.	Case report	An intra-oral scanner was used to obtain patient records, digital photos were taken, and both stump and final shades were selected. To prepare the teeth virtually, these digital records were combined with digital laboratory tools, which then served as chairside templates for guided tooth preparation.	The conventional method relies heavily on the proficiency of the operator to attain an ideal outcome and frequently leads to more destruction of tooth structure. Nevertheless, CAD/CAM technology currently provides a directed method for tooth preparation that minimizes the amount of tooth structure that needs to be removed.	High	Missing clinical data related to the procedures.
[2]	Preparation	To evaluate the accuracy of three digital trial restorations fabricated from digital waxing for ceramic veneers.	In vitro study	Thirty maxillary central incisors were digitally waxed using a standardized technique in a software application. The trial restorations were created using autopolymerizing acrylic resin on typodont teeth. The 30 maxillary central incisors were divided into three groups: the depth cutter group, the round bur group, and the specially designed, calibrated depth-bur group. The three groups were randomly prepared. The aim was to produce an even facial clearance of 0.5 mm.	The trial restoration had a notably greater thickness compared to its equivalent waxing. The DC approach gave the best accurate reduction result among the three protocols studied.	Moderate	No randomization.
[11]	Preparation	To assess the survival of PLVs made with a recently developed, polymer-infiltrated ceramic network material, employing the esthetic pre-evaluative temporary (APT) technique for tooth preparation, and comparing it to the standard technique.	Randomized clinical trial	The study involved 54 laminate veneers administered to six patients in two equal groups: group T, which used the traditional method, and group A, which used the esthetic pre-evaluative temporary procedure. The veneers were evaluated using modified USPHS criteria.	Throughout the study period, veneers were effectively fabricated in both study groups without any instances of secondary caries, endodontic problems, cracks, or loss of retention. No significant fractures were found in any group. Nevertheless, the color-matching standards exhibited a substantial decline in both groups, suggesting a decline in the quality of the veneer. Patients expressed high levels of satisfaction with their veneers. The DC approach yielded the most precise reduction result compared to the other two protocols analyzed.	Moderate	N/A
[5]	Preparation	To assess the accuracy of reduction depths in guided veneer preparation facilitated by four tooth preparation guides.	In vitro study	A total of fifty artificial teeth made of resin were randomly allocated into five distinct groups: a group using freehand technique, a group using a silicone guide, a group using a thermoplastic guide, a group using a 3D-printed guide with uniform stops, and a group using a 3D-printed guide with automatic stopping mechanism. A preparation was done for a window veneer on the upper right central incisor, and the depths of the surfaces that were prepared were measured. Depth maps were generated to assess the accuracy of the decrease depths at each third, employing both veracity and precision measurements.	Tooth preparation guides offer more accuracy for guided veneer preparation as compared to freehand preparation. Out of the four guides, the 3D-printed auto-stop guide had the smallest absolute difference (0.05 mm), whereas the silicone guide had the biggest absolute variation in preparation (0.12–0.16 mm).	High	No cementation guide used.
[1]	Adaptation	To compare between crenelated veneer preparation and conventional preparation in terms of marginal and internal gaps to the prepared tooth surface.	In vitro study	Optical microscopy and micro-CT were employed to assess the marginal and internal gaps in bonded veneers prepared using two methods: crenelated and conventional veneer preparation. Porosity assessments were also conducted.	The novel veneers design produces better marginal and internal adaptation of the restorations to the prepared tooth surface.	High	N/A
[6]	Cementation guide	To describe a digitally designed and 3D-printed cementation guide	Case report	A 3D-printed guide was designed for preparing teeth. This one-piece device can securely hold all veneers together individually, enhancing the stability and precision of the bonding process as compared to using individual holders for each veneer.	This technique offers the ability to precondition and cement multiple veneers simultaneously with increased accuracy, thanks to the application of even and controlled pressure by using flexible resin material during the bonding. It allowed for the use of a rubber dam, resulting in time savings.	High	N/A
[26]	Smile design/Mockup	To investigate the trueness of the mock-ups obtained with milling and 3D-printing technology and a full digital workflow system.	In vivo Study	The study population comprised 10 adult individuals who had DSD (Digital Smile Design) and digital wax-up treatments to improve the esthetic appearance of their upper front teeth. A total of twenty mock-ups were produced, consisting of ten milled mock-ups and ten prototyped mock-ups. STL files were created, and a digital analysis was conducted on the digital wax-up using the surface-to-surface matching approach to evaluate its accuracy. The dimensional parameters of the actual products, the 3D project, and the scanned mock-ups were investigated and compared using specific linear measurements.	Both the prototype and milled mock-ups exhibited a small increase in dimensions compared to the original 3D project. However, the milled mock-ups shown a lower level of fitting after clinical tests. It is important to exercise caution while evaluating the accuracy of scanned manufacturing, because a built-in inaccuracy in the system can result in the underestimation of the thing’s actual dimensions.	Moderate	No randomization
[8]	Preparation	To present a novel digital technology that can be utilized to tackle this difficulty, highlighting its benefits and drawbacks.	Case report	The First Fit system offers a one-step procedure using 3D-printed guides and a custom handpiece for veneer preparation. This method involves creating final restorations and preparing teeth using reduction guides. The veneers are bonded on the same day, while a two-step method involves a preparatory phase using reduction guides and freehand techniques. The process of cementation is then finalized during a second visit.	The suggested technique of guided restorative dentistry employs digital CAD-CAM technology to obtain precise and reliable outcomes in a minimally invasive and efficient manner. The First Fit method utilizes a minimally invasive approach to control and guide veneer preparation. Occasionally, it enables the creation of veneers prior to tooth preparation, thereby eliminating the necessity of temporary restorations.	High	Cannot be generalized to all veneer cases
[4]	Cementation guide	To present a (CAD-CAM) guiding device to facilitate the simultaneous preconditioning and cementation of multiunit porcelain laminate veneers (PLVs).	Technique paper	An intraoral scan was taken preoperatively. The teeth were prepared for veneers and scanned to digitally design the definitive veneers. The veneers were CAD/CAM fabricated and tried in the 3D-printed master model. The veneers and model were desktop-scanned to design the cementation guide digitally. The cementation guide was milled using PMMA. The veneers were preconditioned and loaded using appropriate amount of resin cement using the cementation device.	In contrast with the conventional method, this technique provides a predictable, accurate, and effective way of preconditioning and cementing PLVs.	High	N/A
[27]	Workflow	To describe the clinical protocols for producing PLVs using CAD/CAM technology in a single session, utilizing a digital workflow that includes a facilitating step. Additionally, this report aims to present the clinical outcomes observed over the course of one year.	Case report	Conventional impression → wax-up model → PVS index → Chair-side mockups. Scan of the clinical mock-up + the patient photo → approved 3D smile design and virtual mock-up. Guided tooth preparations were performed based on the approved chair-side mock-up. A 3D smile design was used to create the final veneer designs.	PLV restorations, using CAD/CAM technology, have been efficiently produced, resulting in rapid esthetic rehabilitation and patient satisfaction. This technology ensures precise tooth matching with a minimal margin of error, enhancing the interaction between dentist and patient.	Moderate	No rubber dam isolation or cementation guide used
[28]	Smile design/Mockup	The study compared traditional mock-up production methods to digital workflows, comparing their accuracy to specific designs and diagnostic wax-ups.	In vitro study	The project entailed the fabrication of 52 resin models and the production of a digital depiction of a grin. Both analog and digital wax-ups were acquired, with the analog wax-up utilized for matrices and the digital wax-up employed for PMMA mock-ups. The STL files of the milled prototypes were compared with 3D CAD wax models that were created using a specialized software, while the STL files of the analog-printed prototypes were compared with the conventional wax model design.	The study found that the digital method offers greater accuracy than traditional molded and milled mock-ups, despite the higher risk of errors. This suggests that a fully digital workflow is more reliable for creating esthetic mock-ups, despite the limitations of the study.	Moderate	N/A
[29]	Smile design/Mockup	To demonstrate a methodical approach to diagnose, plan, and stage treatment for a smile makeover by combining DSD with clear aligner therapy. This method successfully accomplishes the intended esthetic design while minimizing the amount of tooth reduction required.	Case report	Phase 1: Digital System Design and Motivational Mockup Phase 2: A digital scan of the inside of the mouth was performed. A treatment plan model was established. Aligners were employed to rectify the front cross-bite, reduce the extraction of healthy tooth tissue, and generate bilateral space around the peg lateral incisors. The photos from each source were overlaid using three reference lines. The therapist subsequently adjusted the software treatment plan to fit with these three guidelines and recorded the Invisalign view for future reference. Phase 3: After aligning the ClinCheck photograph with the DSD, the image of the anticipated Invisalign outcome was overlaid onto the DSD shot. Phase 4: The Invisalign treatment was completed in 8 weeks using a total of 14 aligners. Phase 5: The final restorations were fabricated with e.max.	The integration of Invisalign and DSD facilitated the dentist in repositioning the teeth to optimal locations for veneer restorations, hence attaining the desired aesthetic outcomes with the utilization of less invasive dental procedures. The newly applied veneers seamlessly matched the contours of the face and lower lip, and the gaps were flawlessly sealed. The implementation of the additive technique in this instance reduced the amount of tooth preparation and enhanced the visibility of the teeth in the patient’s smile. The patient expressed satisfaction not only with the exceptional esthetics but also with the little amount of tooth structure that was removed.	Moderate	
[30]	Smile design/Mockup	To describe a digital workflow that employs the virtual smile design approach, augmented with a 3D virtual patient, to restore maxillary central incisors using CAD-CAM fabricated monolithic lithium disilicate ceramic veneers.	Case Report	The 3D facial soft tissue profile of the patient was recorded. The dental laboratory received and imported all digital diagnostic data into CAD software. The 2D and 3D digital data were merged using consistent anatomical landmarks in the soft tissue. A virtual model of facial soft tissue was generated. The STL file containing the 3D intraoral scan was subsequently aligned with the digital representation of the patient. The bespoke 2D virtual grin design served as a visual guide to generate the virtual diagnostic waxing. The abutment teeth were created under the guidance of the vacuum-formed matrix. The creation of definitive veneers involved the use of a machinable lithium disilicate ceramic block.	This clinical report showed that the definitive veneers were fabricated with machinable lithium disilicate ceramic block. No complications were observed during the 6 months after insertion. With the 2D virtual smile design approach, perspective distortion may cause inaccuracy or errors in the conversion process from 2D design to 3D diagnostic waxing, and the use of a 3D virtual patient can overcome this limitation.	Moderate	N/A
[31]	Workflow	To demonstrate the digital process for designing and creating several laminate veneers for a patient’s maxillary anterior teeth, with the aim of improving their aesthetic appearance.	Clinical Report	This clinical report described a cast-free digital workflow for multiple ceramic veneers. Wax-up was performed conventionally. Traditional veneer preparation was performed without using mock-up. Preparation was scanned and margins were defined digitally. Provisional splinted veneers were designed digitally and milled using PMMA.	This procedure improves esthetics by utilizing milled-acrylic temporary restorations and IPS e.max permanent restorations. These restorations meet the clinical standards regarding their fit, form, contour, and esthetics. The utilization of digital dentistry and virtual design has the potential to enhance communication between patients, physicians, and dental laboratories.	High	No rubber dam isolation nore cementation guide
[32]	Preparation	To assess an automated robotic tooth preparation system for PLVs for accuracy and precision compared with conventional freehand tooth preparation	In vitro study	Twenty maxillary central incisor tooth models were divided into two groups: 10 for veneer preparation using a robotic arm and 10 for conventional tooth preparation. All models were scanned, and preoperative design images were superimposed on postoperative preparation images. The dimensional differences between the two images were then measured. The robotic arm was used for veneer preparation in the experimental group.	For data from all sites, the experimental procedure prepared the tooth model with accuracy levels comparable to the control. However, the control procedure demonstrated better precision in tooth model preparation. The experimental group exhibited superior accuracy and precision at the finish line.	Moderate	N/A
[7]	Workflow	To demonstrate the fabrication of an in-office, one-visit CAD/CAM porcelain laminate veneer.	Case report	A porcelain laminate veneer with a fracture was restored in a single visit using CAD/CAM technology. The fractured tooth was first treated with a composite restoration and then scanned. Following the tooth’s preparation, the scanned composite was utilized to design the laminate veneer. The restoration was then milled and cemented.	A single-visit protocol allows clinicians to save time by controlling the color and contour of the porcelain veneer restorations, resulting in a total chair time of 1 h and 45 min.	Low	Missing clinical data of the procedure

**Table 2:** Reasons for article exclusion after full-text reading.

Study	Reason for Exclusion
[33]	Coverage with ceramo-metallic restoration
[34]	Thesis for doctorate
[35]	Composite veneer
[36]	Composite veneer
[37]	Resin bond
[38]	Purely conventional veneers
[39]	Thesis for master’s degree

## Data Availability

No new data were created.

## References

[1] Măroiu A.C., Sinescu C., Duma V.F., Topală F., Jivănescu A., Popovici P.M., Tudor A., Romînu M. (2021). Micro-CT and Microscopy Study of Internal and Marginal Gap to Tooth Surface of Crenelated versus Conventional Dental Indirect Veneers. Medicina.

[2] Luo T., Li J., Xie C., Yu H. (2022). Accuracy of three digital waxing-guided trial restoration protocols for controlling the depths of tooth preparation for ceramic veneers. J. Prosthet. Dent..

[3] (2017). The Glossary of Prosthodontic Terms: Ninth Edition. J. Prosthet. Dent..

[4] Chen X., Zhou N., Ding M., Jing J., Xi Q., Wu G. (2020). A digital guiding device to facilitate cementation of porcelain laminate veneers. J. Prosthet. Dent..

[5] Gao J., He J., Fan L., Lu J., Xie C., Yu H. (2022). Accuracy of Reduction Depths of Tooth Preparation for Porcelain Laminate Veneers Assisted by Different Tooth Preparation Guides: An In Vitro Study. J. Prosthodont..

[6] Silva B.P., Mahn Arteaga G., Mahn E. (2021). Predictable 3D guided adhesive bonding of porcelain veneers using 3D printed trays. J. Esthet. Restor. Dent..

[7] Vafiadis D., Goldstein G. (2011). Single visit fabrication of a porcelain laminate veneer with CAD/CAM technology: A clinical report. J. Prosthet. Dent..

[8] Silva B.P.D., Stanley K., Gardee J. (2020). Laminate veneers: Preplanning and treatment using digital guided tooth preparation. J. Esthet. Restor. Dent..

[9] McLean J.W. (1988). Ceramics in clinical dentistry. Br. Dent. J..

[10] Aristidis G.A., Dimitra B. (2002). Five-year clinical performance of porcelain laminate veneers. Quintessence Int..

[11] Attia Y.S., Sherif R.M., Zaghloul H.H. (2021). Survival of Hybrid Laminate Veneers using two different tooth preparation techniques: Randomized Clinical Trial. Braz. Dent. J..

[12] Calamia J.R. (1985). Etched porcelain veneers: The current state of the art. Quintessence Int..

[13] McLaren E.A. (2013). Bonded functional esthetic prototype: An alternative pre-treatment mock-up technique and cost-effective medium-term esthetic solution. Compend. Contin. Educ. Dent..

[14] Rochette A.L. (1975). A ceramic restoration bonded by etched enamel and resin for fractured incisors. J. Prosthet. Dent..

[15] Sieweke M., Salomon-Sieweke U., Zofel P., Stachniss V. (2000). Longevity of oroincisal ceramic veneers on canines—A retrospective study. Adhes. Dent..

[16] Smales R.J., Etemadi S. (2004). Long-term survival of porcelain laminate veneers using two preparation designs: A retrospective study. Int. J. Prosthodont..

[17] Cattoni F., Mastrangelo F., Gherlone E.F., Gastaldi G. (2016). A New Total Digital Smile Planning Technique (3D-DSP) to Fabricate CAD-CAM Mockups for Esthetic Crowns and Veneers. Int. J. Dent..

[18] Liberati A., Altman D.G., Tetzlaff J., Mulrow C., Gotzsche P.C., Ioannidis J.P., Clarke M., Devereaux P.J., Kleijnen J., Moher D. (2009). The PRISMA statement for reporting systematic reviews and meta-analyses of studies that evaluate healthcare interventions: Explanation and elaboration. BMJ.

[19] Whiting P.F., Rutjes A.W., Westwood M.E., Mallett S., Deeks J.J., Reitsma J.B., Leeflang M.M., Sterne J.A., Bossuyt P.M., QUADAS-2 Group (2011). QUADAS-2: A revised tool for the quality assessment of diagnostic accuracy studies. Ann. Intern. Med..

[20] Balshem H., Helfand M., Schunemann H.J., Oxman A.D., Kunz R., Brozek J., Vist G.E., Falck-Ytter Y., Meerpohl J., Norris S. (2011). GRADE guidelines: 3. Rating the quality of evidence. J. Clin. Epidemiol..

[21] Ahmed W.M., Althagafi R.A. (2023). Smile Makeover Utilizing Digital Esthetic Veneers Workflow: A Case Report. Int. J. Prosthodont. Restor. Dent..

[22] Guzman-Perez G., Jurado C.A., Azpiazu-Flores F., Afrashtehfar K.I., Tsujimoto A. (2023). Minimally Invasive Laminate Veneer Therapy for Maxillary Central Incisors. Medicina.

[23] Li Y., Yu Y., Feng Y., Liu W. (2022). Predictable digital restorative workflow for minimally invasive esthetic rehabilitation utilizing a virtual patient model with global diagnosis principle. J. Esthet. Restor. Dent..

[24] Alshali S., Asali R. (2022). Conventional and Digital Workflow Planning for Maxillary Teeth Restoration with Porcelain Laminate Veneers: A Clinical Report. Clin. Cosmet. Investig. Dent..

[25] Figueira J., Guaqueta N., Ramirez D.I., Kois J. (2023). Veneer tooth preparation utilizing a novel digital designed workflow: A case report. J. Esthet. Restor. Dent..

[26] Lo Giudice A., Ortensi L., Farronato M., Lucchese A., Lo Castro E., Isola G. (2020). The step further smile virtual planning: Milled versus prototyped mock-ups for the evaluation of the designed smile characteristics. BMC Oral Health.

[27] Bayazıt E., Karabıyık M. (2019). Chairside Restorations of Maxillary Anterior Teeth with CAD/CAM Porcelain Laminate Veneers Produced by Digital Workflow: A Case Report with a Step to Facilitate Restoration Design. Case. Rep. Dent..

[28] Cattoni F., Teté G., Calloni A.M., Manazza F., Gastaldi G., Capparè P. (2019). Milled versus moulded mock-ups based on the superimposition of 3D meshes from digital oral impressions: A comparative in vitro study in the aesthetic area. BMC Oral Health.

[29] On Tse R.T. (2019). Merging Clear Aligner Therapy with Digital Smile Design to Maximize Esthetics and Minimize Tooth Reduction. Compend. Contin. Educ. Dent..

[30] Lin W.S., Harris B.T., Phasuk K., Llop D.R., Morton D. (2018). Integrating a facial scan, virtual smile design, and 3D virtual patient for treatment with CAD-CAM ceramic veneers: A clinical report. J. Prosthet. Dent..

[31] Zandinejad A., Lin W.S., Atarodi M., Abdel-Azim T., Metz M.J., Morton D. (2015). Digital workflow for virtually designing and milling ceramic lithium disilicate veneers: A clinical report. Oper. Dent..

[32] Otani T., Raigrodski A.J., Mancl L., Kanuma I., Rosen J. (2015). In vitro evaluation of accuracy and precision of automated robotic tooth preparation system for porcelain laminate veneers. J. Prosthet. Dent..

[33] Mansour F.K., Ibrahim R.M., Mansour H., Hamdy A.M. (2021). Assessment of internal fit and micro leakage of conventionally fabricated ceramometallic restoration versus CAD wax and press veneering (in-vitro study). BDJ Open.

[34] Niranjankumar K. (2020). Survey of Porcelain Laminate Veneers.

[35] Ortensi L., Vitali T., Ortensi M., Lavorgna L., Strocchi M.L. (2020). Customized composite veneers from a totally digital workflow: A case report. Clin. Case Rep..

[36] Revilla-León M., Sánchez-Rubio J.L., Besné-Torre A., Özcan M. (2018). A report on a diagnostic digital workflow for esthetic dental rehabilitation using additive manufacturing technologies. Int. J. Esthet. Dent..

[37] Blatz M.B., Vonderheide M., Conejo J. (2018). The Effect of Resin Bonding on Long-Term Success of High-Strength Ceramics. J. Dent. Res..

[38] Faus-Matoses V., Faus-Matoses I., Ruiz-Bell E., Faus-Llácer V.J. (2017). Severe tetracycline dental discoloration: Restoration with conventional feldspathic ceramic veneers. A clinical report. J. Clin. Exp. Dent..

[39] Guzelian M.C. (2015). Evaluating the Marginal Integrity of Lithium Disilicate Veneers Fabricated by Digital Impressions and CAD/CAM Compared to Conventional Techniques. Master’s Thesis.

[40] Gurrea J., Bruguera A. (2014). Wax-up and mock-up. A guide for anterior periodontal and restorative treatments. Int. J. Esthet. Dent..

[41] LaRue S.E. (2020). Clear Aligner Therapy vs. Traditional Brackets on Smile Arc.

[42] Zhu J., Gao J., Jia L., Tan X., Xie C., Yu H. (2022). Shear bond strength of ceramic laminate veneers to finishing surfaces with different percentages of preserved enamel under a digital guided method. BMC Oral Health.

[43] Da Cunha L.F., Reis R., Santana L., Romanini J.C., Carvalho R.M., Furuse A.Y. (2013). Ceramic veneers with minimum preparation. Eur. J. Dent..

